# Sustainable Super-Sprinkle: Powdered Local Foods

**DOI:** 10.1371/journal.pmed.0020188

**Published:** 2005-07-26

**Authors:** Stacia Nordin

**Affiliations:** **1**LilongweMalawi

I appreciate Zlotkin and colleagues' years of work on the Sprinkle product, and it sounds like the product is much improved from the pill form of micronutrient treatments [1]. I'm not at all opposed to Sprinkle-type products or other nutrient pills for treatment (or in other special situations), but as ten years of international work experience on food and nutrition security issues has shown me, few programmes are supporting local solutions to problems. Once again, a message is being sent that nutrition comes from a pill or packet, made by a foreigner, and requires money.

In the case of Sprinkles, the product could support local solutions by including a message on each sachet about the importance of eating a wide variety of local foods—or a picture of local fruits, vegetables, and legumes. Instead of just sprinkling a packet onto a bulky carbohydrate food, use the Sprinkles as treatment along with instruction about planting and eating less of that bulky carbohydrate in the first place. Even better would be to take all that research, time, energy, and money to teach people (or local manufacturers) how to make their own Sprinkles from local nuts, fruits, greens, oilseeds, insects, fish, and the like.

The results could be just as immediate and dramatic, but with an impact that could last for generations to come. The organisations that support this type of permanent intervention could be mentioned during every teaching session along with big banners and flyers that announce them as the inventors and/or supporters. Just imagine a nice sprinkle powder that everyone can have on hand to improve their own nutrition without relying on a packet from an outside source that is manufactured with machines and jetted in with thousands of litres of petrol (or trucked across the country, if it is made in country).

I'm sure that pre-packaged, imported products have their place in wars, tsunamis, a few cities, and other disasters, but for the majority of the 750 million children in the developing world, their own indigenous foods would have just as much effect, with a longer-term impact on the society's nutritional health.

I saw Zlotkin's presentation on Sprinkles at the International Congress of Dietetics conference in Chicago, Illinois, in 2004, and he did include a sentence about diversifying diets as part of the whole project, but it was strongly overshadowed by discussion of bringing in external resources and experts. When I asked him about using the same resources that went into developing, manufacturing, and transporting Sprinkles to create a local sprinkle product with an emphasis on local diversified diets, he immediately responded that it wouldn't work.

How do we know, if no one really puts the effort into it at the level that products like Sprinkles get?

I've posted this message to several food and nutrition listservs and magazines, and I am now beginning to learn of some small projects working towards local sprinkle products. Zlotkin and team could assist these projects to research the work and scale it up to other countries with other local foods.
